# Supplementary material for: A symbiotic physical niche in Drosophila melanogaster regulates stable association of a multi-species gut microbiota
Source: Nat Commun. 2023 Mar 21;14:1557. doi: 10.1038/s41467-023-36942-x (PMC10030875; doi:10.1038/s41467-023-36942-x)
Supplement: Supplementary file 1 — Supplementary Information [file 41467_2023_36942_MOESM1_ESM.pdf]

Supplementary Information for

A symbiotic physical niche in *Drosophila melanogaster* regulates stable association of a multi-species gut microbiota

Authors: Ren Dodge[1], Eric Jones[2,3], Haolong Zhu[1,4], Benjamin Obadia[5], Daniel J. Martinez[1], Chenhui Wang[1,6], Andrés Aranda-Díaz[7], Kevin Aumiller[1,4], Zhexian Liu[4], Marco Voltolini[8], Eoin L. Brodie[8], Kerwyn Casey Huang[7,9,10], Jean Carlson[3], David Sivak[2], Allan C. Spradling[1,4,6], William B. Ludington[1,4*]

Correspondence to: ludington@carengiescience.edu

This PDF file includes:

Supplementary Text
Figs. S1 to S12
Caption for Movie S1

Other Supplementary Materials for this manuscript include the following:

Movie S1
SourceData_Dodge_etal_2023.zip

Supplementary Text

Ai exhibits increased early death rates in germ-free flies

To probe the facilitation of *Ai* colonization by *LpWF*, we examined the dynamics of *Ai* colonization from 1 hpi to 6 dpi (Fig. S3G,H, S6B,C,E-N, S11). For the first 1 dpi, *Ai* abundance was significantly higher in *LpWF*-pre-colonized versus germ-free flies (Fig. 3A, S6G). After 2 dpi, *Ai* levels were only slightly higher in *LpWF*-pre-colonized flies (Fig. S6G). Thus, the presence of *LpWF* ameliorates the initial decrease in *Ai* levels, which could stem from a decrease in the growth rate, an increase in the death rate or the egestion rate, or some combination of these factors. We comprehensively measured each of these rates.

We measured growth rate in the fly using fluorescent protein plasmid dilution due to growth in the absence of antibiotic selection (Fig. S6B,C,E-I)[1]. The mean generation time of *Ai* was similar in initially germ-free and *LpWF*-pre-colonized flies (0.21 vs. 0.23 h^{-1}, Welch's t-test, p= 0.75; Fig. S6I). However, the variance in plasmid loss was significantly higher in germ-free flies compared with *LpWF*-pre-colonized flies (F-test, p=0.014), consistent with the observed population bottleneck (Fig. S6G), which we also previously observed in certain *Lp* strains and connected to a population bottleneck shortly after inoculation[1]. Thus, different growth rates of *Ai* cells with or without *LpWF* do not seem to account for the differences in *Ai* abundance.

To determine whether the initially germ-free flies egested *Ai* cells more rapidly than *LpWF*-pre-colonized flies, we measured the egestion rate from the abundance of *Ai* in their frass (excrement) after 1 h in a fresh vial. The rate of viable *Ai* egested by initially germ-free flies reached zero by 1 dpi, while *Ai* egestion in *LpWF*-pre-colonized flies remained higher and never reached zero (Fig. S11). Differences in egestion rate could be due to more rapid passage through the fly or to variable death rates of the bacteria inside the fly. To measure rates of passage

through the fly, we fed fluorescent polystyrene beads simultaneously with *Ai* inoculation, and the proportion of egested beads was quantified over time by flow cytometry (Fig. S6K). The rate of bead egestion was highly similar between *LpWF*-pre-colonized and germ-free flies (Fig. S6K). Thus, transit time through the gut does not explain the differences in *Ai* colonization dynamics, suggesting a higher death rate of the *Ai* cells colonizing an initially germ-free gut.

Since egestion is tightly linked to ingestion[2], we measured the total *Ai* consumed by flies versus that remaining in the vial after feeding by counting CFUs in flies and on the food 1 hpi, reasoning that any bacteria not accounted for must have died during the 1 h of feeding (Fig. S6, S12), e.g. by lysis in the digestive tract. In both sets of flies, only a small fraction of the inoculum was left 1 hpi (Fig. S1, S12). These measurements indicate that germ-free and *LpWF*-pre-colonized flies consumed the same amount of *Ai* and that *Ai* has a higher survival rate in the gut of *LpWF*-colonized flies.

The higher survival in co-colonized guts could be due to bacterial interspecies interactions, such as a cytoprotective effect of *LpWF* on *Ai*, or to host-microbe interactions, such as the fly gut becoming more hospitable to *Ai* when pre-colonized by *LpWF*. To differentiate between these two possibilities, we fed germ-free flies with *LpWF* and *Ai* simultaneously, reasoning that host priming would not be evident with simultaneous colonization (Fig. S6M,N). *Ai* abundance at 1 hpi in co-inoculated flies was similar to initially germ-free flies fed *Ai* alone, and significantly lower than in *LpWF*-pre-colonized flies 1 hpi (Fig. S6N), indicating that *LpWF* remodels the host in a manner beneficial to *Ai*. We also measured *Ai* survival 1 hpi when colonizing *Ai*-pre-colonized flies. A slight advantage was observed (Fig. S6M), which was substantially less than for *Ai* colonizing *LpWF* flies (c.f. Fig. S3G). *In vitro, Ai* abundance was unaffected by co-culturing with *LpWF*[3]. Because the *Ai* cells are alive in the proventriculus but

dead upon defecation, a simple explanation consistent with our data is that for *Ai* cells colonizing *LpWF*-pre-colonized flies, more *Ai* cells are retained for a longer period of time in the proventriculus, and cells that are not retained in the proventriculus die when passing through the midgut. Taken together, our results indicate that the host environment is more permissive to *Ai* survival when pre-colonized by *LpWF*.

Supplementary Figures (S1 through S12) follow:

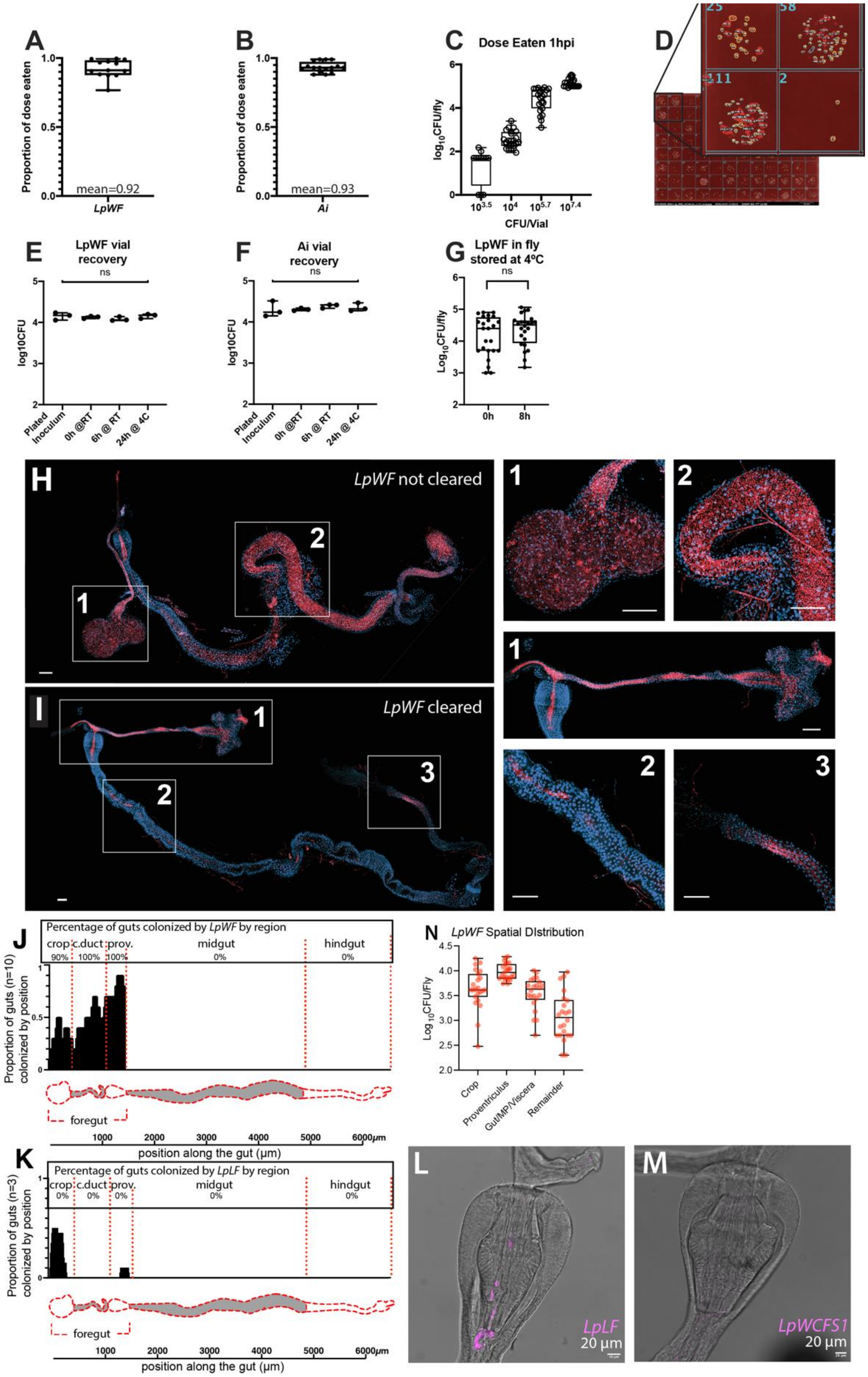

Figure S1. Validation of colonization assay and culturing techniques.

A. *LpWF* dose consumed was assayed by washing the food in 1x PBS and plating the solution. A dose of $10^{3.5}$, 10^4, or $10^{5.7}$, $10^{7.4}$ CFUs/vial was fed on top of agar food in standard vials. Flies ate >90% of the dose after 1 hour (n=12 vials, mean=0.9235). Results were normalized to the dose. The proportion of the dose consumed was calculated by subtracting the leftover inoculum from the delivered inoculum and normalizing to the delivered inoculum. The growth of bacteria on the food over the 1 hour feeding window was monitored by using a parallel control vial that did not have flies added (see panel E).
B. *Ai* dose eaten: flies ate >90% of dose after 1 hours (n=16 vials, mean=0.93). Same methods as panel A.
C. CFU abundance in flies 1 hpi. Flies were inoculated by feeding on standard food, 25 flies/vial. For doses $10^{3.5}$, 10^4, $10^{5.7}$, and $10^{7.4}$ CFUs/vial (equal to $10^{2.1}$, $10^{2.6}$, $10^{4.2}$, and 10^6 CFUs/fly respectively), flies all ate a similar amount of bacteria. For the lowest dose, $10^{3.5}$ CFUs total in the vial, which was about 125 CFU/fly, 3 of 12 flies sampled had 0 detectable CFUs 1 hpi. $n \geq 24$ flies/dose. The limit of detection was 50 CFUs.
D. For CFU quantification, flies were collected into 96 well plates containing 100µl PBS and 0.1µm glass beads. In our standard assays, CFUs were quantified by spotting 2µl of the 100 µL fly homogenate (in 96 well plates) onto growth media in rectangular tray plates so that each well of the 96 well plate was spotted. Microcolonies were grown for 30 h at 30˚C. Counting was performed by photographing plates, counting colonies in ImageJ, and manually validating. Because the maximum amount of homogenate plated is 1/50th of a fly, a count of 1 colony yields a value of 50 CFUs/fly; the *resolution* of this quantification system is 50 CFUs, which we also call the limit of detection (LOD). To distinguish the invading strain from the resident strain in the priority effects experiments, invading bacteria containing a resistance plasmid were used and plated on selective media, CFU quantification in GF control flies was done in parallel during the same experiment using also the same plasmid-containing inoculum and counted on the same selective media.
E. Validation experiment shows that the number of CFUs recovered did not vary significantly from the inoculum measured by directly plating. Bacteria were recovered from vials by rinsing with 2 mL PBS then plating a dilution to count CFUs. Inoculum was recovered immediately after placing on the fly food, after leaving at room temperature for 6 hours, and storing at 4ºC overnight. *LpWF* bacteria were used. 3 independent vials per treatment. One-way ANOVA; mean of each column was compared with the mean of every other column using Dunnett's multiple comparisons test.
F. Validation of *Ai* recovery from vials was the same as in D, CFU counts were consistent for *Ai*. 3 independent vials per treatment. One-way ANOVA; mean of each column was compared with the mean of every other column using Dunnett's multiple comparisons test.
G. When flies could not be homogenized and plated immediately, they were stored at 4ºC for up to 8 h. To test for any possible effects on the bacterial abundance, flies from the same vial were homogenized either immediately or after storage for 8 h at 4ºC (n=23 flies/time point). There was no significant difference in CFU counts. (n=46, unpaired t-test p=0.2794)

H. Transient microbes are found throughout the gut in flies kept on the same food for more than 24 h. *LpWF* is labeled with mCherry (red). Note the distended crop (1) and food filled midgut (2), the punctate appearance of the mCherry indicates bacteria dispersed throughout the fly food. Blue is a single z-slice of DAPI stain to indicate the gut boundary. Scale bars 100 µm.

I. Guts were cleared of transient microbes by placing on agar-water starvation media for 3 hours. *LpWF* remains in the foregut (1), the esophageal tract is lined with a dense and continuous population of *LpWF*, whereas there is a patchy appearance in the crop. mCherry signal is largely absent from the midgut aside from a few small patches (2), and it is absent from the hindgut, although some autofluorescence occurs (3). Scale bars 75 µm.

J. Quantification of spatial distribution of *LpWF* in the fly digestive tract. Mean intensity of mCherry fluorescent signal in *LpWF*-mCherry-colonized flies 3-5 dpi, n=10 guts was converted to percentage of colonized guts per region (top) and the proportion of colonized guts as a function of the normalized distance along the gut tract. Smoothing was performed by spatial averaging. Drawing depicts a segmentation of an average gut oriented lengthwise beginning with the crop. Quantification is explained in the Methods.

K. Quantification of spatial distribution of *LpLF* in the fly digestive tract as in J. n=5 guts.

L. Microscopy of *LpLF* in the proventriculus shows lower colonization than for *LpWF*. Scale bar 20 µm.

M. *LpWCFS1* shows very low abundance in the proventriculus. Scale bar 20 µm.

N. Raw CFU counts of spatial distribution of *LpWF* in dissected gut regions (Fig. 1G) shows the majority of CFUs in the fly gut are in the proventriculus and crop duct. n=24 biological replicates dissected into 4 segments each.

Box and whiskers plots: center of box is median; box encloses 25th to 75th percentiles; whiskers indicate minimum and maximum.

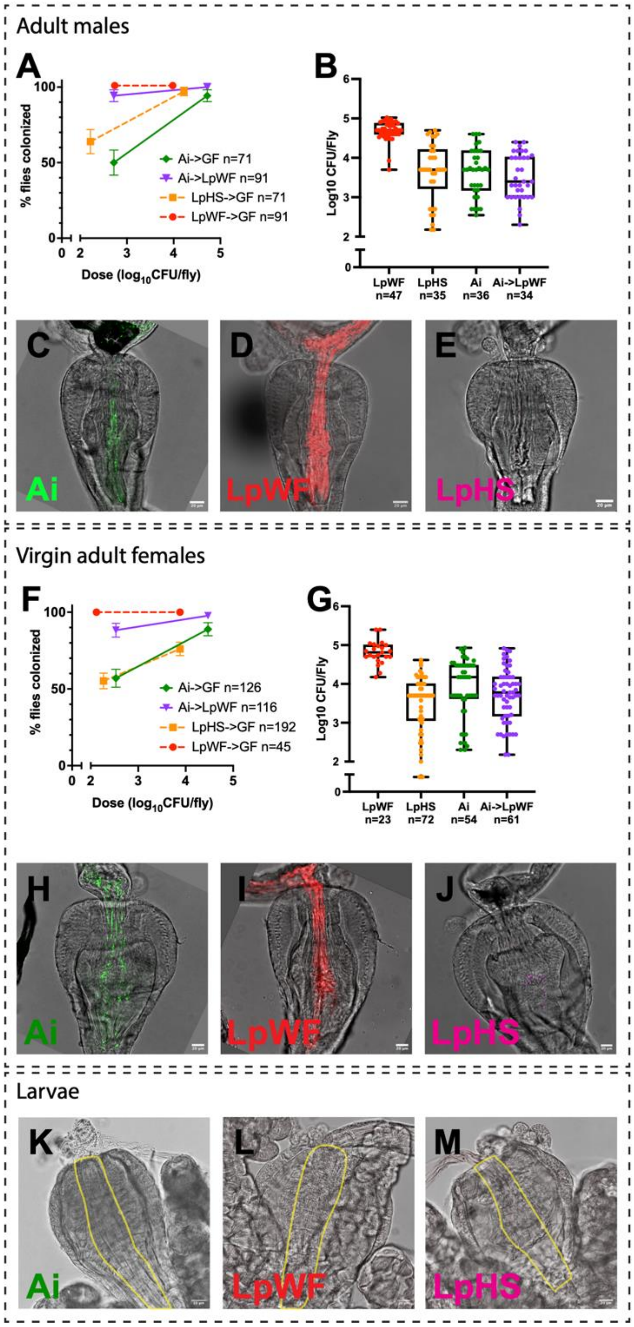

Figure S2. The niche is present in adult males and virgin females but not larvae.

A. Dose response in adult male flies; *Ai* fed to germ-free flies (*Ai*->GF), *LpWF* fed to germ-free flies (*LpWF*->GF), *Ai* fed to flies colonized with *LpWF (Ai->LpWF)*, *LpHS* fed to germ-free flies (*LpHS*->GF); n≥71 individual flies in 3 biological replicates; error bars show standard error of the percentage (SEP).
B. Steady state abundance in adult male flies (n=36 flies, >3 biological replicates).
C. Max intensity Z-projection of *Ai* colonization in adult male proventriculus.
D. Max intensity Z-projection of *LpWF* colonization in adult male proventriculus.
E. Max intensity Z-projection of colonization *LpHS* in adult male proventriculus.
F. Dose response in virgin female flies; n≥45 individual flies in 3 biological replicates; error bars are SEP.

G. Steady state abundance in virgin female flies (n=72, >3 biological replicates).
H. Max intensity Z-projection of *Ai* colonization in adult virgin female proventriculus.
I. Max intensity Z-projection of *LpWF* colonization in adult virgin female proventriculus.
J. Max intensity Z-projection of *LpHS* colonization in adult virgin female proventriculus.
K. Max intensity Z-projection of *Ai* colonization in larval proventriculus. Yellow line marks inner lumen.
L. Max intensity Z-projection of *LpWF* colonization in larval proventriculus. Yellow line marks inner lumen.
M. Max intensity Z-projection of *LpHS* colonization in larval proventriculus. Yellow line marks inner lumen. Scale bars are 20 μm.

Box and whiskers plots: center of box is median; box encloses 25th to 75th percentiles; whiskers indicate minimum and maximum.

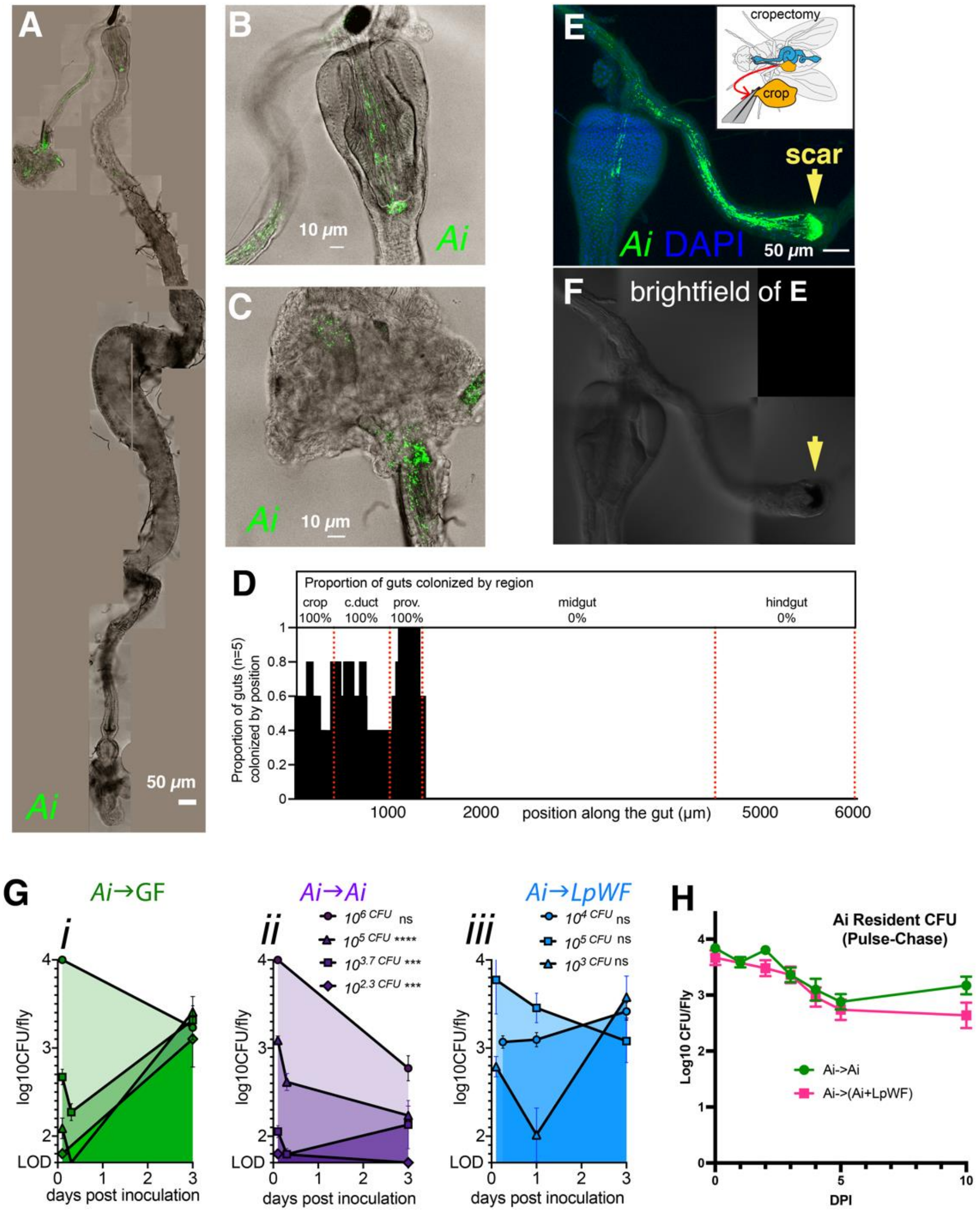

Figure S3. *Ai* colonization is similar to *LpWF* colonization.

A. Whole mount gut colonized by *Ai*-mGFP5.
B. Detail of proventriculus.
C. Detail of crop.
D. Spatial quantification of colonization as in Figure S1J.
E. *Ai* colonization of the foregut after cropectomy surgery as in Fig 1. Green = *Ai*-mGFP5. Blue = DAPI. Scale bar 50 μm. n=14 of 14 flies colonized after cropectomy surgery. Yellow arrowhead indicates melanization at site of crop duct severing.
F. Brightfield image of the foregut in E.
G. Time course bacterial abundance for *Ai* colonizing (i) germ-free, showing convergence on a carrying capacity (ii) *Ai*-colonized, showing exclusion by resident *Ai*, and (iii)

LpWF-colonized flies, showing no exclusion. Data points are mean of $\log_{10}$ CFUs abundance/individual fly; n=24 individual flies per data point; error bars are SEM.

H. Pulse-chase of *Ai* into *Ai*-mGFP5-pre-colonized flies (green) or flies pre-colonized by *Ai*-mGFP5 and *LpWF* (pink), indicating slow turnover of the resident *Ai* cells with a half-life of ≈2.5 d and a plateau of ~1,000 CFUs. Data points show mean of $\log_{10}$ CFUs in individual flies over a 10 day period. $n \geq 45$ individual flies/data point with ≥3 biological replicates; error bars represent SEM.

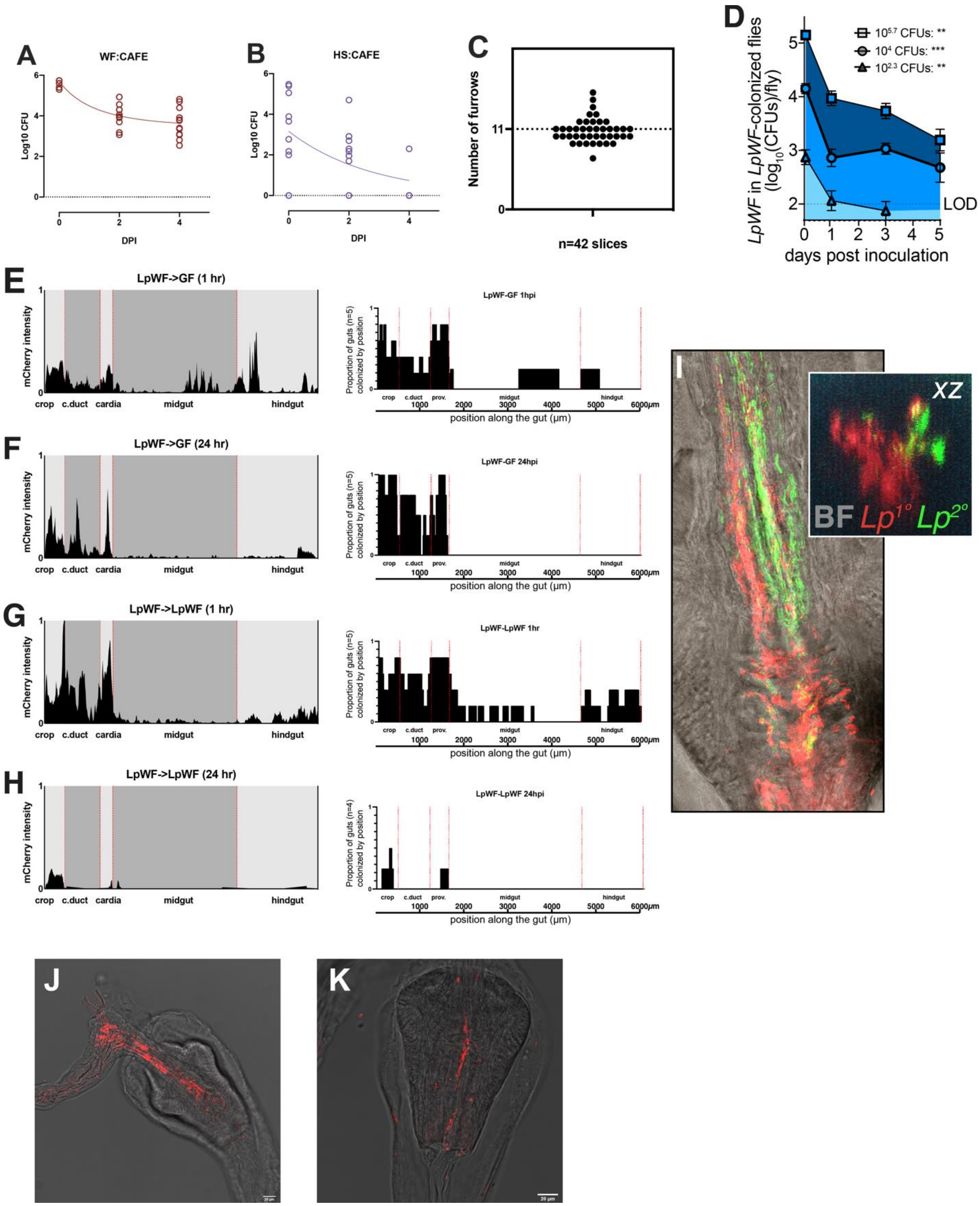

Figure S4. Timecourses of microbe populations demonstrate a low turnover rate for *LpWF*.

A. *LpWF* High into GF flies transferred daily to a fresh vial with only CAFÉ-supplied liquid food (10% glucose, 5% yeast extract, 0.42% propionic acid) over 5 dpi.

B. *LpWCFS1* High into GF flies transferred daily to fresh vial with only CAFÉ-supplied liquid food (10% glucose, 5% yeast extract, 0.42% propionic acid) over 5 dpi.
C. The number of furrows in the proventriculus, mean=10.89. Furrows were counted in 42 TEM images from various points along the length of the proventriculus. *n*=5 different proventriculi.
D. A single dose of *LpWF*-mCherry was fed at a range of doses (see inset) to flies pre-colonized by *LpWF* and *LpWF*-mCherry CFUs were quantified over 5 d, indicating the abundance does not converge at ~10^4 CFUs/fly as when the doses are fed to initially germ-free flies (c.f. Fig. 2A). Data points show mean of $\log_{10}$ CFUs in individual flies over a 5 day period; $n \geq 24$ flies/timepoint with ≥3 biological replicates; error bars represent SEM.
E. (E-H) Quantification of spatial distribution of *LpWF* along the gut. LEFT PANELS: Mean intensity of *LpWF*-mCherry fluorescence fed to either GF or *LpWF* pre-colonized flies at 1 hour or 24 hours after inoculation. Summed intensity projections of 80-µm thick stacks of confocal images of whole gut dissections were quantified for fluorescence intensity, normalized to total intensity and length. RIGHT PANELS: Proportion of guts colonized as a function of the normalized distance along the gut tract (c.f Fig. S1J, S2D). N=4 or 5 flies per treatment. E: *LpWF*-mCherry → GF at 1 hpi.
F. *LpWF*-mCherry → GF at 24 hpi.
G. *LpWF*-mCherry → *LpWF* at 1 hpi.
H. *LpWF*-mCherry → *LpWF* at 24 hpi.
I. *LpWF*-sfGFP → *LpWF*-mCherry 1 hpi of *LpWF*-sfGFP. Confocal fluorescent image of proventriculus. Inset: optical x-z cross section. Note that we typically did not observe the secondary colonizer in the furrows, but when we did, the cells of the secondary dose clustered tightly.
J. *LpWF*-mCherry in the proventriculus during pulse-chase experiment 3 d after initiation of unlabeled *LpWF* chase. Note reduced colonization versus Fig. 1.
K. *LpWF*-mCherry in the proventriculus during pulse-chase experiment 5 d after initiation of unlabeled *LpWF* chase. Note reduced colonization versus panel J of this figure.

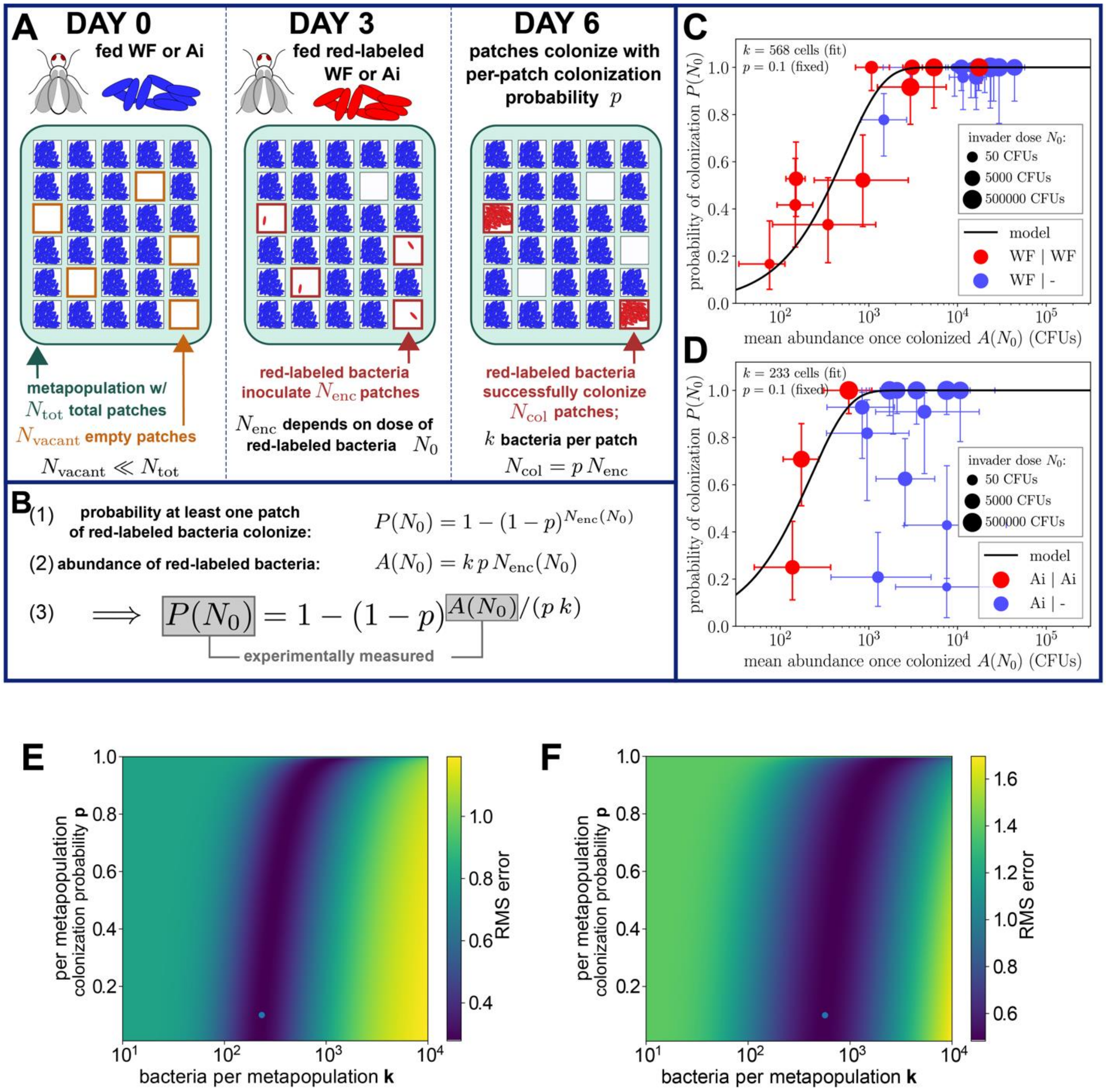

Figure S5. Quantitative model of colonization relates population turnover to invadability.

A. Model describes the spatial variability of bacterial colonization with a metapopulation model of patchy colonization, assuming that the fly gut may be subdivided into N_{tot} subpopulations based on the observation that turnover occurs on the time scale of 15 d. On day 0 a strong colonizer (*LpWF* or *Ai*, colored blue) is fed to the fly. By day 3, the initially fed blue bacterial species are assumed to colonize the majority of the patches, leaving N_{vacant} patches uncolonized. When colonized, each patch has a carrying capacity of k bacteria. On day 3 a red-labeled but otherwise identical bacteria (*LpWF* or *Ai*, colored red) is fed to the fly at an abundance N_0, and the red-labeled bacteria proceed to inoculate some N_{enc} of these patches; with probability p these inoculated patches become fully colonized with k bacteria, and with probability $1 - p$ they go extinct by day 6.

B. Equation describing the model. (1) The probability of invader colonization as a function of the dose. (2) Abundance (A) of invader in terms of the per-patch carrying capacity k, the per-patch probability of colonization p, and the number of inoculated patches N_{enc}. Eliminating N_{enc} yields the third equation. (3) Relationship between the experimentally measurable probability of colonization P_{col} and the invader abundance A. The two free parameters p and k may be fit; these parameters have the biological significance of indicating how bacteria are distributed among patches when colonizing, thus informing their spatial distribution.

C. Consistent with the model, $LpWF \rightarrow LpWF$ priority effect experiments show a positive correlation between mean abundance $A(N_0)$ and probability of colonization $P(N_0)$, and when fit to the metapopulation model with $p = 0.1$ fixed predicts the per-patch carrying capacity k to be 568 cells. Each data point consists of a number of biological replicates, ranging from n=4 to n=28. X error bars are 95% confidence intervals of the mean, computed by bootstrapping. Y error bars are 95% confidence intervals of the proportion, computed using the Jeffreys interval.

D. $Ai \rightarrow Ai$ priority effect experiments predict a per-patch carrying capacity of 233 cells. Error bars show 95% confidence intervals. Error bars same as Fig. S5C.

E. Error probability function for the fit of k to the $LpWF$ data shows that the fit of k is robust.

F. Error probability function for the fit of k to the Ai data shows that the fit of k is robust.

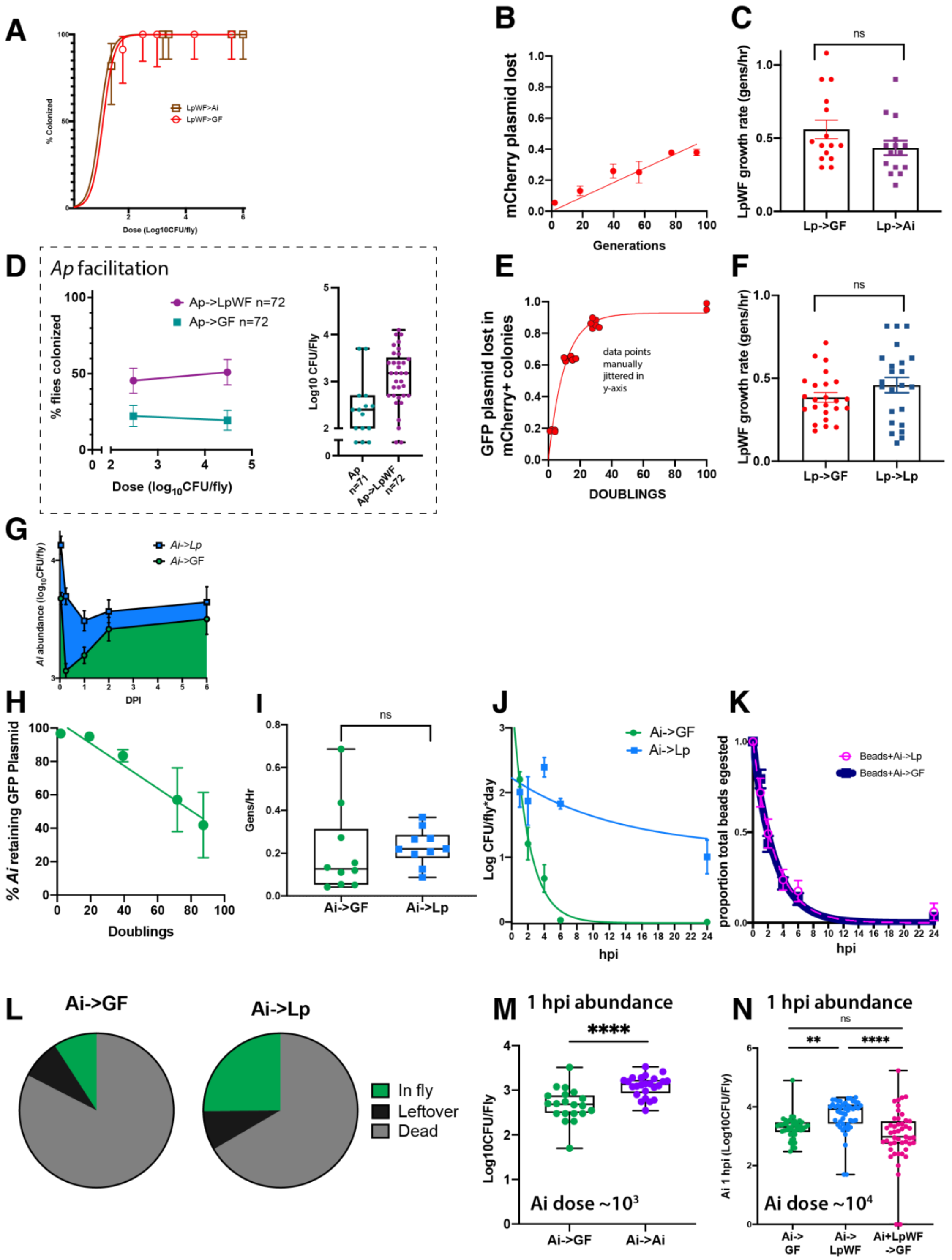

Figure S6. Dose response and kinetics in vivo.

A. Dose response for *LpWF* fed to *Ai*-pre-colonized flies. *n*=24 flies/dose; error bars represent the 95% confidence intervals (Jeffries method).
B. Plasmid loss standard curve for pCD256NS-P1-mCherry-ΔEc (mCherry-Cam) in *LpWF* enumerated daily for 5 d by plating on non-selective media and counting fluorescent vs. non-fluorescent colonies. 100-fold daily dilution in 3 mL culture. Slope of the simple

linear regression of *in vitro* plasmid loss rate was 0.004624 of total colonies per doubling event (R^2=0.07301). *n*=3 biological replicates for each point; error bars represent SEM.

C. Growth rate of *LpWF in vivo*. *LpWF* invading GF flies had a mean growth rate 0.4589 gen/hr 5 d after invasion while *LpWF* invading *Ai* flies grew at a lower but not significantly different mean of 0.3849 gen/hr (n=15 individual fly homogenates per condition; columns height indicates mean growth rate; error bars show SEM; Welch's two-tailed t-test, p=.1272; CF-test no significant difference in variances, p=0.3499). The ~2-fold variation in individual fly measurements is expected due to a population bottleneck that we previously characterized [1].

D. Left: Dose response for *Acetobacter pasteurianis (Ap)* in germ-free adult mated females (*Ap*->GF) and in adult mated females colonized with *LpWF* (*Ap->LpWF*). Right: Steady state abundance in *Ap* colonized adult mated female flies. *n*=72 individual flies ≥3 biological replicates; error bars represent SEM.

E. Dual plasmid standard curve: plasmid loss in *LpWF* containing both plasmids pCD256NS-P11-mCherry-ΔEc and pTRKH2-mGFP5 (GFP-Erm) was measured as a ratio of colonies positive for GFP-Erm plasmids (which are lost rapidly) divided by those positive for mCherry-Cam (which is retained much longer). This standard was modeled as an exponential function with a plateau: $y = 1-(0.9326*\exp(-0.07325*x))$, R^2=0.9986. n=6 biological replicates per time point; note that replicates points are overlapping.

F. Growth rate of *LpWF* invading *LpWF* pre-colonized flies. *LpWFCam/Erm* invading GF flies had a mean growth rate 0.4589 gen/hr, whereas *LpWFCam/Erm* invading *LpWF* pre-colonized flies had a mean of 0.5596 gen/hr as estimated from CFUs in flies 5 dpi with *LpWFCam/Erm*. There was no significant difference in growth rates (Welch's two-tailed t-test, p=0.1768). An F-test to compare variances was significant (p=0.034) where *LpWF* invading *LpWF* pre-colonized flies had a higher variance in plasmid loss, suggesting a founder effect due to lower initial population. *n*≥24 flies per sample; *n*≥3 biological replicates.

G. *Ai* CFU abundance over time comparing flies germ-free at 0 dpi with flies pre-colonized by *LpWF* at 0 dpi. *Ai* abundance is lower in GF flies vs in flies pre-colonized by *LpWF* at 1 hpi, 6 hpi, and 1 dpi (*p*<0.0001, independent, unpaired Welch's two-tailed t-tests, Bonferroni correction) but not at 2 dpi or 5 dpi (p>0.05). Data points show mean $\log_{10}$ CFUs abundance in individual flies over a 5 day period, n≥43 flies/data point; *n*≥3 biological replicates; error bars represent SEM.

H. *Ai* plasmid loss standard curve: Growth in the absence of antibiotic selection leads to plasmid loss that is correlated with the number of cell divisions. The ratio of colonies with:without plasmid pCM62-mGFP5-tet (GFP-Tet) in *Acetobacter indonesiensis* SB003 was quantified daily for 5 d by plating on non-selective media and counting fluorescent vs. non-fluorescent colonies as a function of the total amount of culture growth. The slope of the linear regression of this standard curve was 0.56% percent of cells lost their plasmid every doubling event. This rate was applied to plasmid loss by bacteria in flies to estimate the *in vivo* growth rate. Percentage of plasmid was measured daily for 5 d. Y = 0.005579*x, (R^2=0.1590)

I. Mean growth rate 6 d after inoculation was 0.2287 generations per hour (gen/hr) for *Ai* invading *LpWF* pre-colonized flies or 0.2060 gen/hr for Ai invading GF flies (n=10 samples of 8 flies each). There was no significant difference in growth rates between *Ai*

growth rate in flies (Unpaired Welch's two-tailed t-test, p=0.7528). Higher variance was observed for *Ai* invading GF flies (F-test, p=0.014).

J. Transit time of *Ai* through the gut to GF or *LpWF*-pre-colonized flies in the first day after inoculation. *Ai* was fed along with polystyrene beads to flies (dose = 1.2 x 10^5 CFUs of *Ai*/fly) in standard food in the cap of a 50 mL Falcon tube. *Ai* shedding was measured by counting CFUs recovered from falcon tubes by rinsing with PBS then centrifuging the contents to concentrate bacteria and beads for flow cytometry. Half-life of *Ai* in GF during the first day was 1.5 hours, while egestion of *Ai* in *LpWF* never decayed to zero. n=8/treatment; error bars represent SEM.
K. Shedding of 0.5-μm fluorescent, polystyrene beads co-fed to flies with *Ai* in FIG 5C. Beads were counted by flow cytometry. (~4 x 10^5 beads fed per fly). Half-life of beads was 1.9 or 2.0 hours in GF flies vs. in *LpWF* pre-colonized flies respectively, a non-significant difference (95% CI of decay fit). *n*=16 (beads+*Ai*→GF), *n*=8 (beads+*Ai*→*Lp*); error bars represent SEM.
L. Proportion of *Ai* dose remaining in vials after feeding, viable in flies, or killed, n=12 vials per condition. Proportions are normalized among 3 groups of flies fed doses of 3.0 x 10^3, 3.0 x 10^4, and 3.2 x 10^5 CFU/fly.
M. Number of live CFUs of *Ai* in flies 1 hpi comparing *Ai* into GF flies vs *Ai* into flies pre-colonized by *Ai*. Dose was ~10^4 CFUs/fly. n=20 flies/condition. ****: p<0.0001.
N. Number of live CFUs of *Ai* in flies 1 hpi comparing *Ai* alone into GF flies versus *Ai* alone into *LpWF*-pre-colonized flies versus *Ai*+*LpWF* mixed into GF flies. Dose was 3 x 10^4 CFUs of *Ai*/fly; *n*=48 flies/condition. For *Ai*+*LpWF* mixed, dose of *LpWF* was 3 x 10^4 CFUs/fly; *n*=47 flies condition. ****: p<0.0001, **: p=0.0055.

   Box and whiskers plots: center of box is median; box encloses 25th to 75th percentiles; whiskers indicate minimum and maximum.

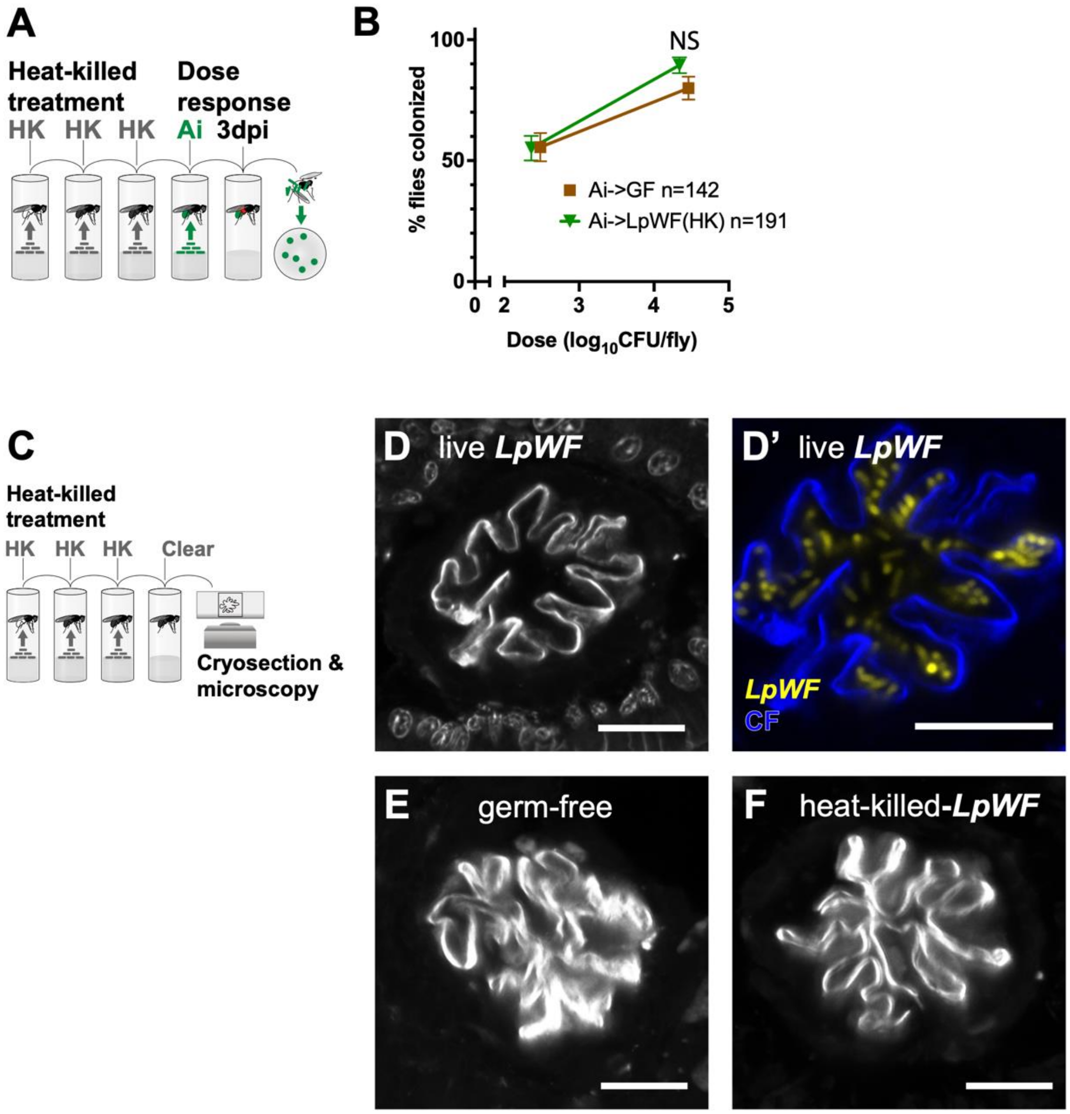

Figure S7. Heat-Killed *Lactobacillus plantarum* (*LpWF*) does not elicit host-response

A. Schematic of priority effects experiment in heat-killed treated flies; flies were fed heat-killed LpWF as in (A), then fed doses of *Ai* bacteria.
B. Dose response for *Ai* in germ-free and *Ai->LpWF* in heat-killed treated flies (n=72 flies).
C. Schematic of heat-killed experiment; to mimic colonization by *LpWF*, 5-7do mated female flies were fed with heat-killed LpWF bacteria daily for 3 days, the gut was then cleared of excess killed bacteria by transferring to fresh food overnight and then to agar-water for 4 hours before embedding and freezing for cryosectioning.
D. Cross section of anterior proventriculus in *LpWF* colonized flies stained with calcofluor. Furrows are expanded by presence of bacteria. D'. Color rendering of D with bacteria shown. yellow = *LpWF*-mCherry bacteria, blue=cuticle stained with calcofluor.
E. Cross section of germ-free anterior proventriculus stained with calcofluor. Furrows are more narrow than colonized.
F. Heat-killed treated anterior proventriculus stained with calcofluor. Furrows are not significantly enlarged following 3 days feeding with *LpWF*.
All scale bars are 10 µm.

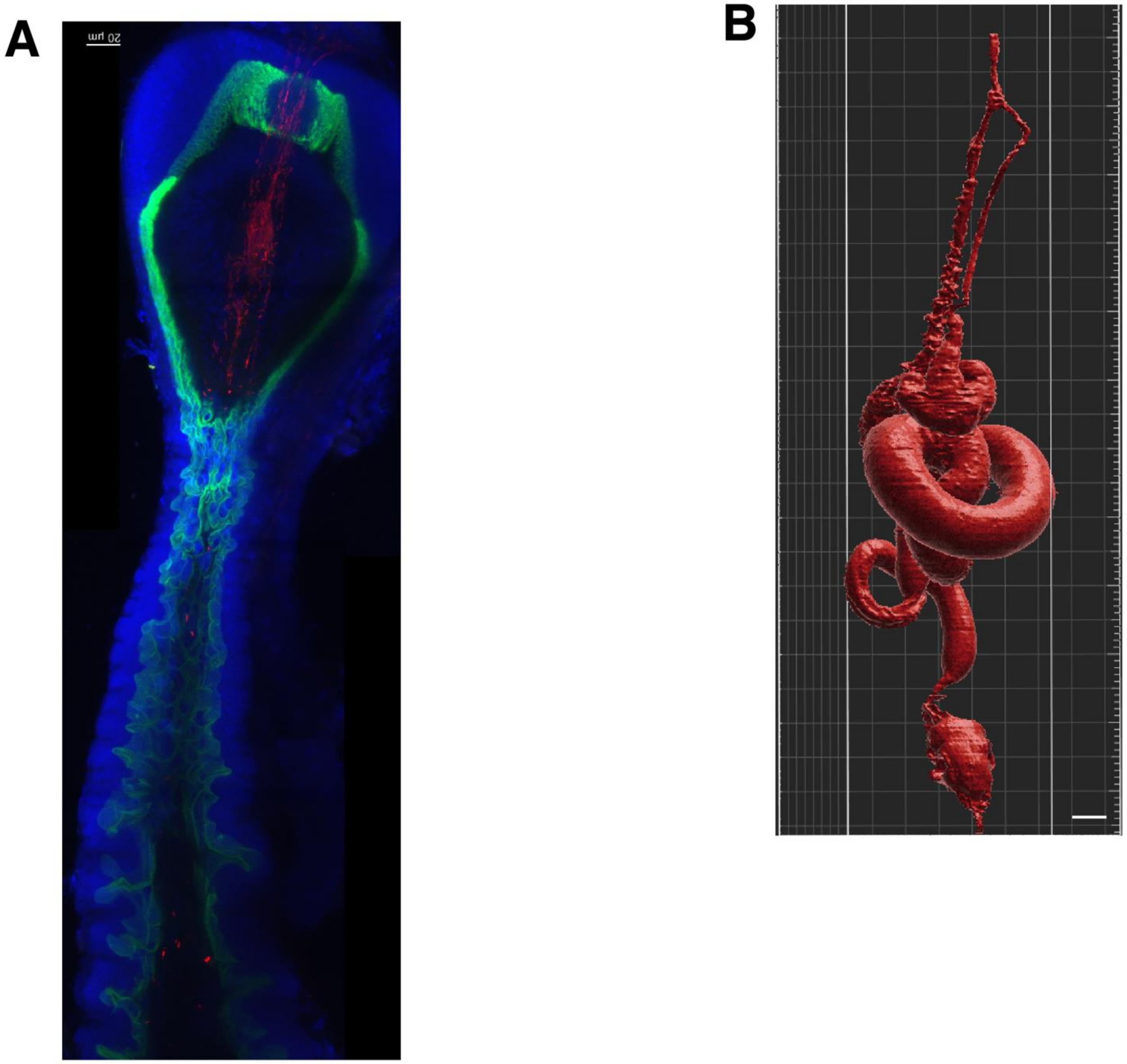

Figure S8. Imaging crypt spaces.

A. Foregut colonized by LpWF-mCherry in A142-GFP brush border reporter transgene flies (generously provided by the Buchon Lab) shows no overlap between the brush borders in the outer proventriculus lumen and the colonization in the inner lumen. Brush borders (green), *LpWF*-mCherry (red), DNA/DAPI (blue). Scale bar = 20µm.

B. Whole fly gut model made using XR-µCT, as in Fig. 4A-F. Used to compute volume of the 3 segments assayed in Fig. 1F. Segment volume: Foregut: $5.08\text{x}10^{6}$ µm^{3}, Midgut: $4.60\text{x}10^{7}$ µm^{3}, Hindgut: $6.45\text{x}10^{6}$ µm^{3}, Cardia: $3.39\text{x}10^{5}$ µm^{3}, Crop: $4.75\text{x}10^{6}$ µm^{3}, Visera: $5.24\text{x}10^{7}$ µm^{3}. Rough surfaces in the volume rendering correspond to crypts that are visualized by the brush border marker in A.

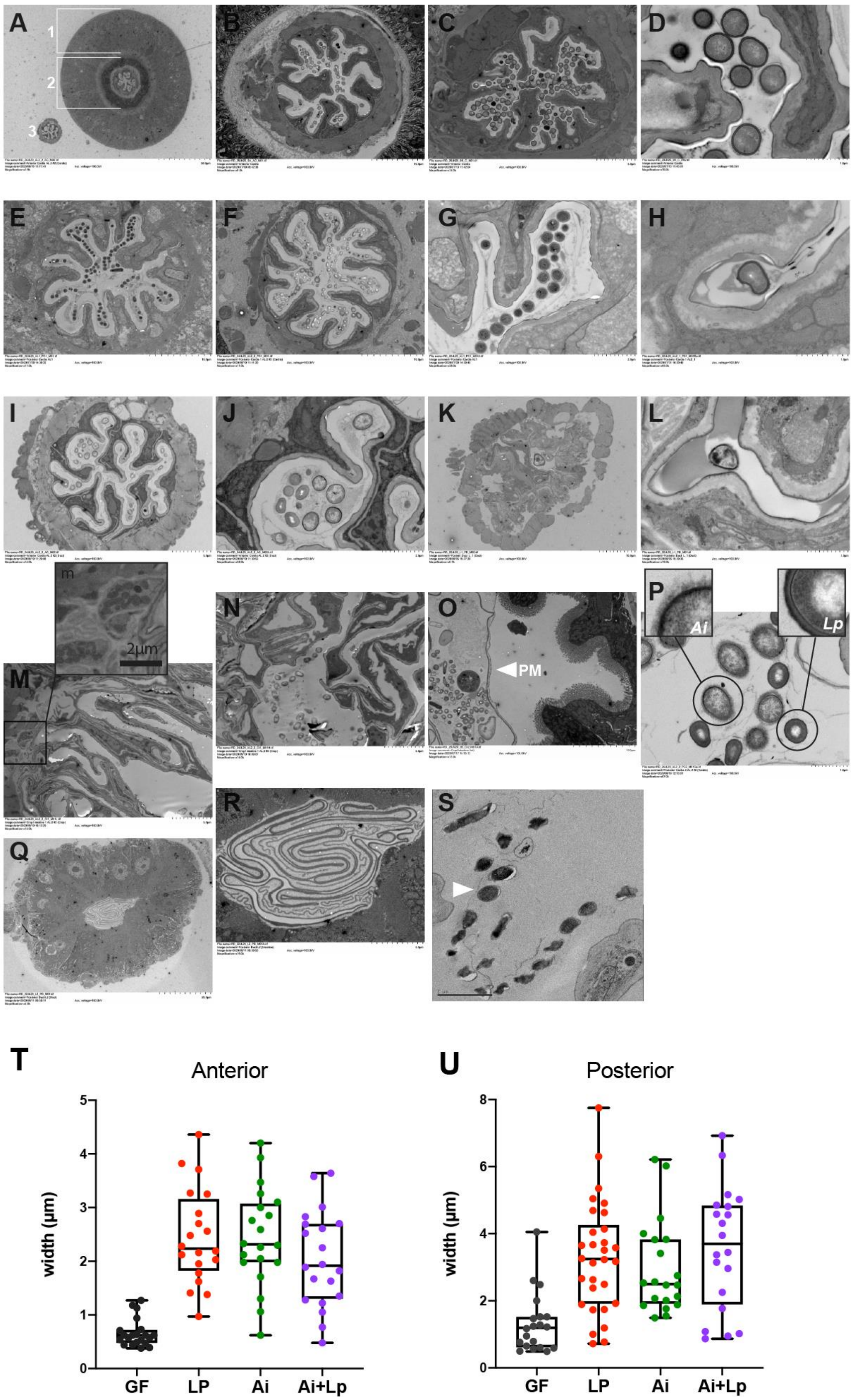

Figure S9. Spatial Structure of Colonization by Transmission Electron Microscopy

A. Overview of the anterior proventriculus. The mesodermal, midgut portion of the proventriculus (the proventriculus or outer proventriculus) is indicated (1). The ectodermal, foregut portion (the inner proventriculus or stomadeal valve) in indicated (2). The crop duct is present in this section as well (3). (*Ai+LpWF* colonized)
B. Anterior proventriculus post feeding (*LpWF* 1 hpi)
C. Anterior proventriculus post feeding (*LpWF* 1 hpi)
D. *LpWF* packed in anterior proventriculus furrow (*LpWF* 1 hpi)
E. Posterior proventriculus colonized (*Ai->LpWF* 1 hpi
F. Posterior proventriculus colonized (*Ai+LpWF* 5 dpi)
G. Posterior proventriculus furrow, (*Ai->LpWF* 1 hpi). Only *LpWF* visible.
H. Long narrow furrow with single *Lp* cell (*Ai+LpWF* colonized)
I. Crop Duct, similar morphology to proventriculus. (*Ai+LpWF* colonized)
J. Detail of crop duct in I (*Ai+LpWF* colonized)
K. Posterior crop duct/anterior crop, sparsely colonized (*LpWF* colonized)
L. Single bacterium in posterior crop duct (*LpWF* colonized)
M. Crop wall cuticle. Inset: cluster of bacteria. (*Ai+LpWF* Colonized)
N. Crop lumen and cuticle (*Ai+LpWF* Colonized).
O. Midgut, bacteria are separated from the brush borders (BB) by the peritrophic membrane (PM) (LpWF 1 hpi).
P. Posterior proventriculus: both *Ai and LpWF* in the lumen of the posterior proventriculus. The gram negative *Ai* can be identified by a fuzzy coat (the glycocalyx or fimbriae) and its larger size relative to *LpWF*. *LpWF* is gram positive, it is distinguished by its think cell wall. (*Ai+LpWF* colonized)
Q. Constriction between posterior proventriculus and anterior midgut, where the peritrophic matrix (PM) is extruded from proventriculus outer lumen (*LpWF* colonized).
R. PM immediately posterior to the proventriculus (*LpWF* colonized).
S. High pressure freezing shows cleared zone between the lumen wall and bacteria, indicating the boundary region shown in Fig. 4M is not a fixation artefact.
T. Quantification of proventriculus furrow width in the anterior proventriculus for germ-free flies and flies colonized with *LpWF*, *Ai*, or *Ai+LpWF*. n=2 proventriculi per treatment and 10 sections per proventriculus.
U. Quantification of proventriculus furrow width in the posterior proventriculus for germ-free flies and flies colonized with *LpWF*, *Ai*, or *Ai+LpWF*. n=2 proventriculi per treatment and 10 sections per proventriculus.
   Box and whiskers plots: center of box is median; box encloses 25th to 75th percentiles; whiskers indicate minimum and maximum.

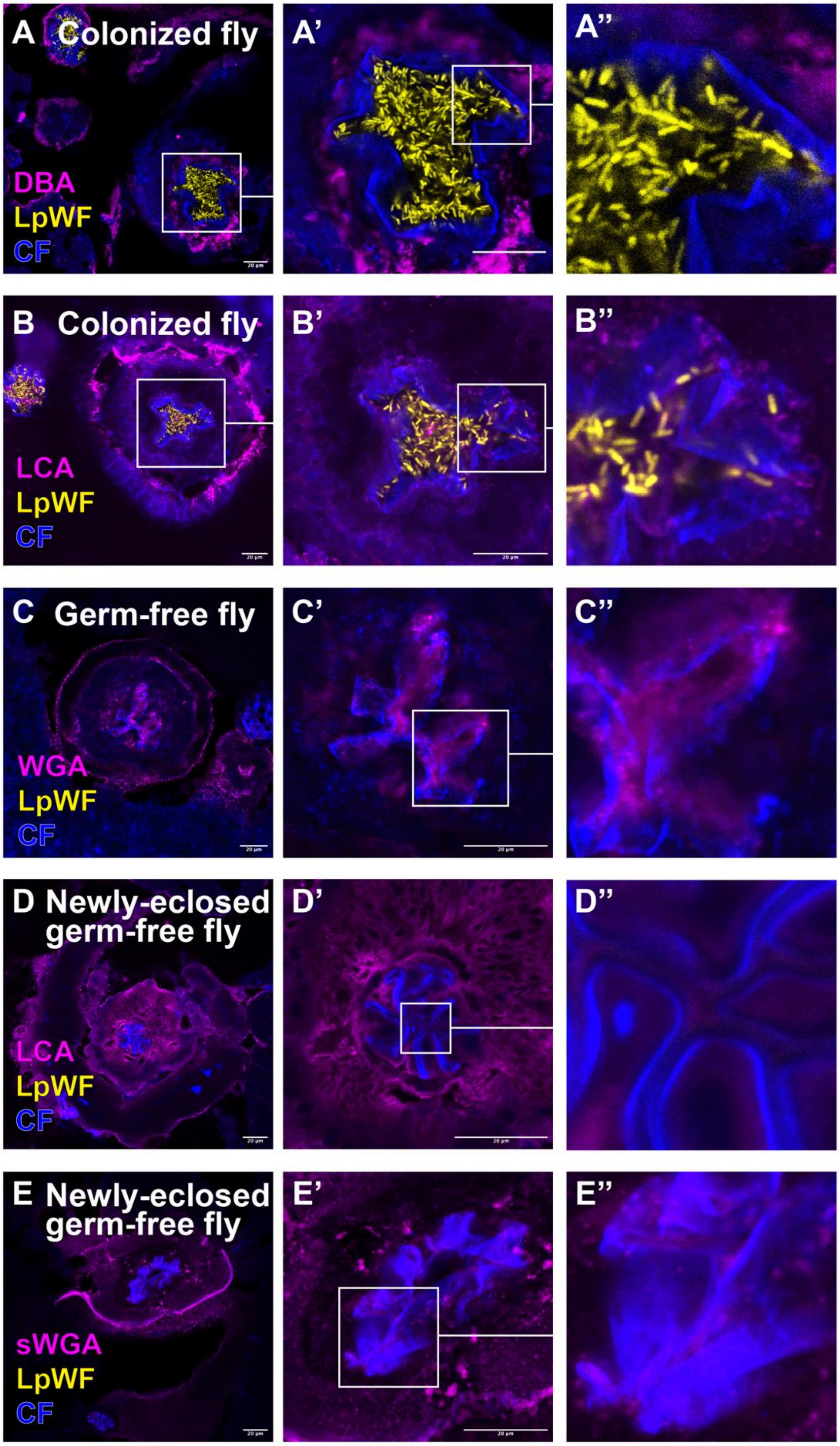

Figure S10. Lectin staining of the proventriculus.

A. DBA staining a transverse cross section of a colonized proventriculus.

B. LCA staining a transverse cross section of a colonized proventriculus.
C. WGA staining a transverse cross section of a germ-free proventriculus.
D. LCA staining a transverse cross section of a newly eclosed germ-free proventriculus.
E. sWGA staining a transverse cross section of a newly eclosed germ-free proventriculus.
Scale bars are 20 µm.
n≥3 biological replicates per treatment.

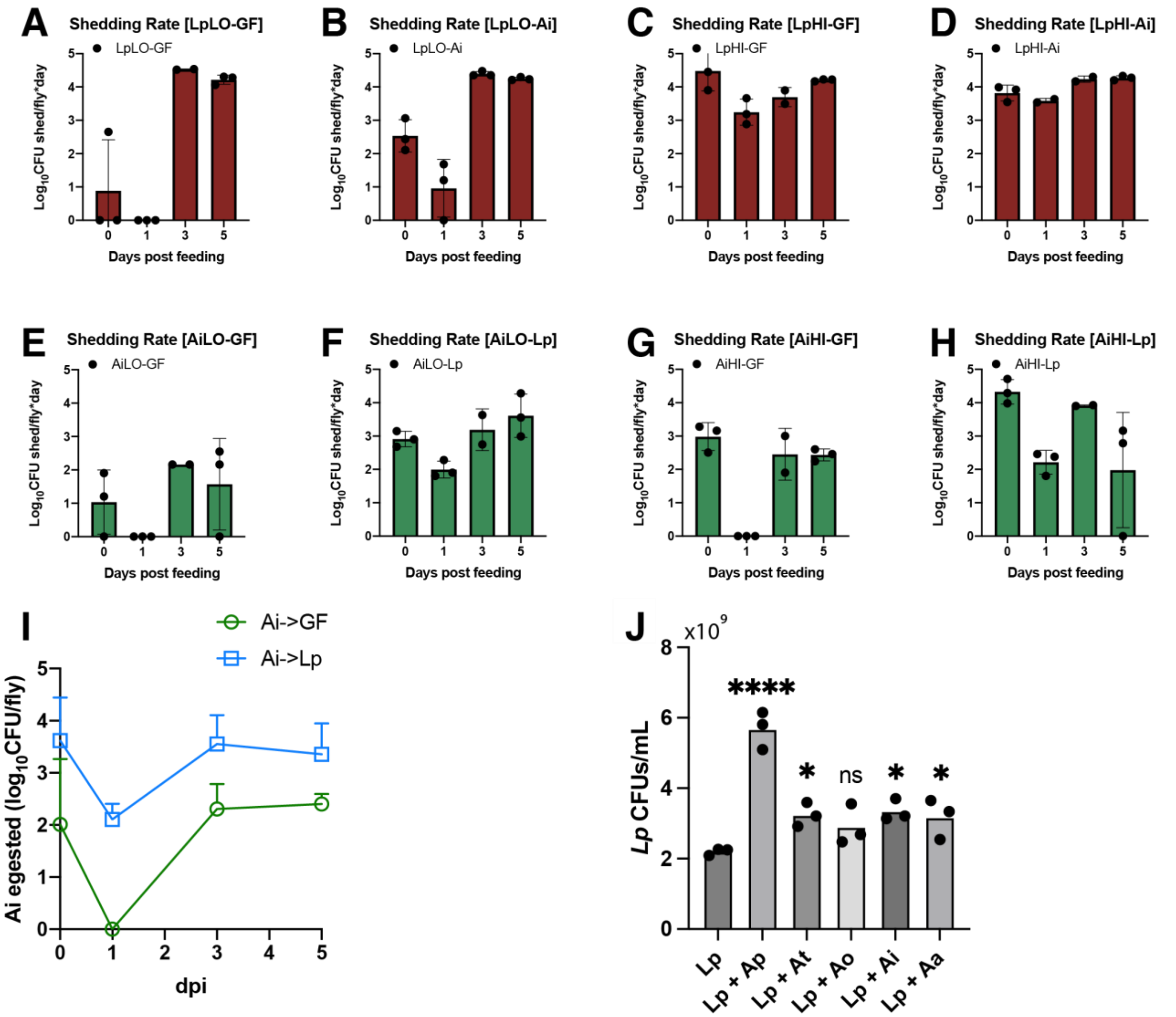

Figure S11. Egestion of bacteria by flies following inoculation.
Shedding rates for various conditions following inoculation with bacteria were measured by keeping flies in a vial for a period of 1 hour, recovering viable bacteria from the vial by rinsing with PBS, then plating to count CFUs. Treatments correspond to the same experiments as in figures S5A-S5H. <u>Doses:</u> *LpWF* Low: 2.0 x 10^3 CFUs/fly; *LpWF* High: 5.7 x 10^5 CFUs/fly. 12 flies were sampled daily and analyzed for CFU counts.

A. *LpWF* Low into GF flies. *n*=3 independent vials/time point; columns indicate mean of $\log_{10}$ CFU abundance/individual fly; error bars show standard deviation.
B. *LpWF* Low into flies pre-colonized by *Ai*.
C. *LpWF* High into GF flies.
D. *LpWF* High into flies pre-colonized by *Ai.* (A-D) Regardless of dose, *LpWF* egestion rate was lowest 1 dpi, suggesting a period of establishment. 3 dpi, *LpWF* CFUs are shed at a consistent rate of 2 x 10^4 CFU/fly/day, about equal to the stable population of LpWF (Fig S5A).
E. *Ai* Low into GF flies. 3 biological replicate vials.

F. *Ai* Low into flies pre-colonized by *LpWF*. 3 biological replicate vials.
G. *Ai* High into GF flies. 3 biological replicate vials.
H. *Ai* High into flies pre-colonized by *LpWF*. (E-G) *Ai* shedding rate is variable over time and between treatments. 3 biological replicate vials.
I. Combined Low and High inoculations from E-H plotted on same graph. After 24 hours, the average number of *Ai* egested reaches 0 in GF flies then increases to a mean of 2.5 x 10^2 CFU/fly/hour. The number of egested *Ai* in *LpWF*-pre-colonized flies is significantly higher at all time points, never drops to 0, and achieves an average rate of 3.2 x 10^3 CFU/fly/day.
J. Co-culturing *Lp* with *Ap*, *At*, *Ai*, or *Aa* resulted in increased *Lp* cell density after 48 h. Co-culturing with Ao did not significantly increase *Lp* cell density by 48 h. *n*=3 per condition. One-way ANOVA; P-values are from a Student's two-sided t-test of the difference from the monoculture (****: P<0.0001, *: P<0.05). (reproduced from [3])

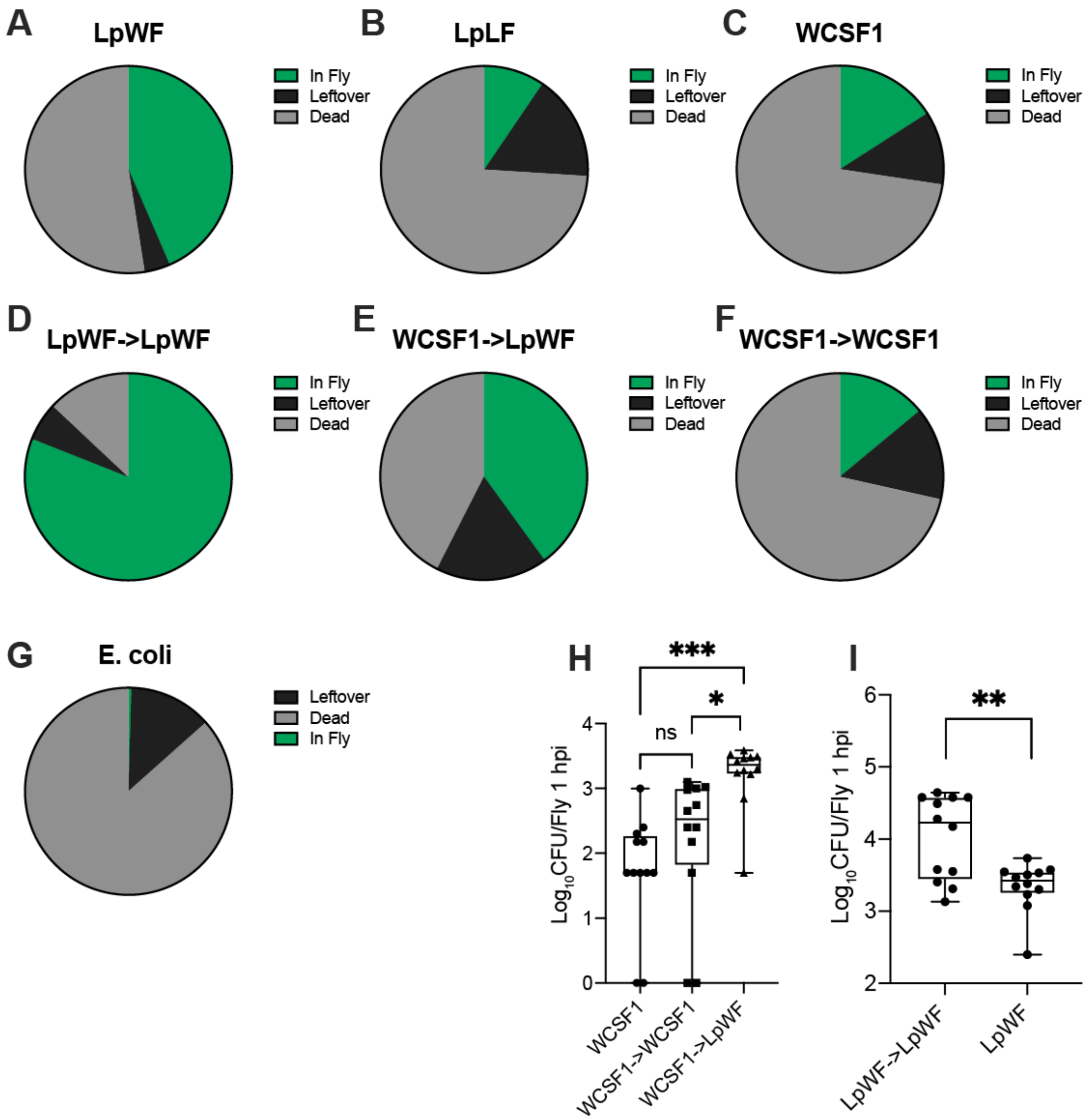

Figure S12. Survival and death of *Lp* strains following inoculation

A-F: The proportion of viable bacteria in the fly 1 hour post inoculation was measured alongside the bacteria remaining in the vial (leftovers), these numbers were subtracted from initial dose placed in the vial to estimate the number of bacteria killed. Proportions used for pie charts were calculated on a per fly basis. Values for flies that were fed doses of ~10^5 and ~10^7CFU/vial were combined because we did not observe significant difference (n=24 flies/bacterial strain combined from 2 vials of 12 flies/strain). The proportion of bacteria consumed (1 minus the leftover fraction) varies between strains, indicating that *LpWF* is more readily consumed by flies. These measurements were used to calculate the per-fly dose in the experiments and adjust the dose accordingly. Limit of detection = 50 CFUs.

A. *LpWF* fed to germ-free flies.
B. *LpLF* fed to germ-free flies.
C. *WCSF1 (LpHS)* fed to germ-free flies.
D. *LpWF* fed to flies pre-colonized with *LpWF*.

E. *WCSF1* fed to flies pre-colonized with *LpWF*.
F. *WCSF1* fed to flies pre-colonized with *WCSF1*.
G. *E. coli* JM110 fed to germ-free flies.
H. CFU surviving in flies fed a dose of WCSF1 (2 x10^5 CFU/vial or 1 x 10^4 CFU/fly, n=12 flies). Survival of *WCSF1* after one hour was significantly higher in flies pre-colonized with *LpWF* (p=0.0006, one-way ANOVA). Survival of *WCSF1* in flies pre- colonized with *WCSF1* was not significantly higher. Survival of invading *LpWF* dose was better in flies pre-colonized with *LpWF* (4 x 10^4 CFU/vial or 3 x 10^3 CFU/fly, n=12 flies). p=0.0020, one-way ANOVA).
I. CFU surviving in flies fed a dose of *LpWF* 2 x10^5 CFU/vial or 1 x 10^4 CFU/fly, n=12 flies). Survival of *LpWF* after one hour was significantly higher in flies pre-colonized with *LpWF* (*p*=0.002, unpaired, two-sided t-test).
Box and whiskers plots: center of box is median; box encloses 25th to 75th percentiles; whiskers indicate minimum and maximum.

Supplementary References

1. Obadia, B. *et al.* Probabilistic Invasion Underlies Natural Gut Microbiome Stability. *Curr. Biol.* **27**, (2017).
2. Wu, Q. *et al.* Excreta Quantification (EX-Q) for Longitudinal Measurements of Food Intake in Drosophila. *iScience* **23**, 100776 (2020).
3. Aranda-Díaz, A. *et al.* Bacterial interspecies interactions modulate pH-mediated antibiotic tolerance. *Elife* **9**, (2020).
