# Supplementary material for: A symbiotic physical niche in Drosophila melanogaster regulates stable association of a multi-species gut microbiota
Source: Nat Commun. 2023 Mar 21;14:1557. doi: 10.1038/s41467-023-36942-x (PMC10030875; doi:10.1038/s41467-023-36942-x)
Supplement: Supplementary file 3 — Description of Additional Supplementary Files [file 41467_2023_36942_MOESM3_ESM.pdf]

Description of Additional Supplementary Files

File Name: Supplementary Movie 1
Description: Lp-Ai colonization. 3d rendering of Lp and Ai co-colonizing the niche
